# Cholinergic Depletion in Alzheimer's Disease Shown by [^**18**^F]FEOBV Autoradiography

**DOI:** 10.1155/2013/205045

**Published:** 2013-11-10

**Authors:** Maxime J. Parent, Marc-Andre Bedard, Arturo Aliaga, Luciano Minuzzi, Naguib Mechawar, Jean-Paul Soucy, Esther Schirrmacher, Alexey Kostikov, Serge G. Gauthier, Pedro Rosa-Neto

**Affiliations:** ^1^Translational Neuroimaging Laboratory, McGill Centre for Studies in Aging, Douglas Mental Health University Institute, Montreal, Canada H4H 1R3; ^2^Département de Psychologie, Université du Québec à Montreal (UQAM), Montreal, Canada H3C 3P8; ^3^Department of Psychiatry, McGill University, Douglas Mental Health University Institute, Montreal, Canada H4H 1R2; ^4^Montreal Neurological Institute (MNI), Montreal, Canada H3A 2B4

## Abstract

*Rationale*. Alzheimer's Disease (AD) is a neurodegenerative condition characterized in part by deficits in cholinergic basalocortical and septohippocampal pathways. [^18^F]Fluoroethoxybenzovesamicol ([^18^F]FEOBV), a Positron Emission Tomography ligand for the vesicular acetylcholine transporter (VAChT), is a potential molecular agent to investigate brain diseases associated with presynaptic cholinergic losses. *Purpose*. To demonstrate this potential, we carried out an [^18^F]FEOBV autoradiography study to compare postmortem brain tissues from AD patients to those of age-matched controls. *Methods*. [^18^F]FEOBV autoradiography binding, defined as the ratio between regional grey and white matter, was estimated in the hippocampus (13 controls, 8 AD) and prefrontal cortex (13 controls, 11 AD). *Results*. [^18^F]FEOBV binding was decreased by 33% in prefrontal cortex, 25% in CA3, and 20% in CA1. No changes were detected in the dentate gyrus of the hippocampus, possibly because of sprouting or upregulation toward the resilient glutamatergic neurons of the dentate gyrus. *Conclusion*. This is the first demonstration of [^18^F]FEOBV focal binding changes in cholinergic projections to the cortex and hippocampus in AD. Such cholinergic synaptic (and more specifically VAChT) alterations, in line with the selective basalocortical and septohippocampal cholinergic losses documented in AD, indicate that [^18^F]FEOBV is indeed a promising ligand to explore cholinergic abnormalities *in vivo*.

## 1. Introduction

Alzheimer's Disease (AD) is the most prevalent neurodegenerative condition in individuals over 65 [1]. AD neuropathology is characterized by progressive accumulation of amyloid beta peptide (brain amyloidosis), intraneuronal inclusion of hyperphosphorylated tau (neurofibrillary tangles), and degeneration of various neuronal populations. Depletion of cholinergic cell bodies located in the basal forebrain constitutes one of the earliest findings observed in postmortem AD brains [2]. This forebrain neurodegeneration depletes cholinergic projections in virtually the entire prosencephalon. Specifically, degeneration of the Nucleus Basalis of Meynert as well as the medial septum severely perturbs cholinergic neurotransmission at the level of the cortex and hippocampus, respectively [3]. In contrast, cholinergic neurotransmission remains mostly unaffected in other brain regions such as the basal ganglia, thalamus, brainstem, and cerebellum.

Quantification of presynaptic terminals provides significant information regarding numerous biochemical processes related to cholinergic neurotransmission. For example, acetylcholine synthesis and transport into vesicles occur in the presynaptic terminals by the action of choline acetyltransferase (ChAT) and the vesicular acetylcholine transporter (VAChT), respectively. VAChT availability has been proposed as an excellent indicator of presynaptic integrity [4], which is decreased in AD.

The neurobiological basis of cholinergic vulnerability in AD is unknown; however, a link between amyloid pathology and cholinergic depletion has been supported in animal models of AD [5, 6]. In AD patients, reduced cholinergic presynaptic terminals are associated with amyloid burden [7]. In contrast, upregulation of ChAT in the hippocampus of populations at risk for AD suggests cholinergic sprouting or acetylcholine synthesis upregulation as a response to cholinergic cell depletion [8]. Overall, the degeneration of cholinergic basal forebrain projections has been consistently shown to strongly correlate with clinical symptoms [9, 10]. As such, efficient surrogate markers of presynaptic cholinergic integrity are potential tools for the assessment of AD, as well as other neurodegenerative diseases where cholinergic systems are compromised.

Imaging of cholinergic presynaptic innervation has been made possible by the introduction of molecular probes selective for VAChT derived from vesamicol [11] such as [^18^F]Fluoroethoxybenzovesamicol ([^18^F]FEOBV). In contrast to previous VAChT molecular agents, [^18^F]FEOBV has shown desirable kinetic properties, high selectivity for VAChT, and low affinity for sigma receptors [12]. Positron Emission Tomography with [^18^F]FEOBV has been successfully performed in primates [13] and in rodent models to quantify cholinergic declines secondary to aging or selective immunolesions [14].

Quantification of VAChT depletion in AD using [^3^H]vesamicol autoradiography has not been very conclusive [15, 16], most likely due to the low specificity and affinity of that ligand [4]. Given that [^18^F]FEOBV has a much higher specificity than [^3^H]vesamicol, we aimed in the current study to assess its potential as a marker of pathological cholinergic degeneration, by comparing brain tissues of AD patients to those of healthy controls using [^18^F]FEOBV autoradiography. Demonstration of significant differences could constitute useful *postmortem* evidence in support of future clinical *in vivo* applications of [^18^F]FEOBV.

Given that both neocortex and hippocampus are the main targets of cholinergic projections known to be compromised in AD, [^18^F]FEOBV binding in these regions was hypothesized here to be significantly lower in AD than in control tissues. 

## 2. Materials and Methods

Frozen brain samples from healthy controls and AD-positive brains (diagnosis confirmed by neuropathological reports; CERAD criterion) were obtained from the Douglas-Bell Canada Brain Bank (Douglas Mental Health University Institute, Montreal, Canada). Utilization of these samples was approved by both the Douglas Institute's research ethics board and the Brain Bank's scientific review committee. Samples were excluded in cases of psychiatric conditions, epilepsy, non-AD dementia, brain neoplasms, and traumatic brain injuries. In total, 21 samples from the hippocampus (13 controls, 8 AD) and 24 from the prefrontal cortex (PFC) (13 controls, 11 AD) were dissected from the left hemispheres. Using a freezing sliding microtome (Leica CM3050 S) at −15°C, each block of tissue was cut into serial 20 *μ*m-thick sections, which were then thaw-mounted on coated microscope slides. Two adjacent sections from each sample were randomly selected and respectively used for [^18^F]FEOBV autoradiography and for histological staining, as described below, allowing to precisely localize regions of interest (ROIs). 

[^18^F]FEOBV was synthesized based on a method described previously [17, 18]. Since only the (−)-[^18^F]FEOBV enantiomer shows affinity to VAChT [12], a pure *levo* precursor (*ABX advanced biochemical compounds GmbH*) was used to ensure optimal affinity. Labeling with [^18^F] was done using a GE TRACERlab module.

**Figure 1:**
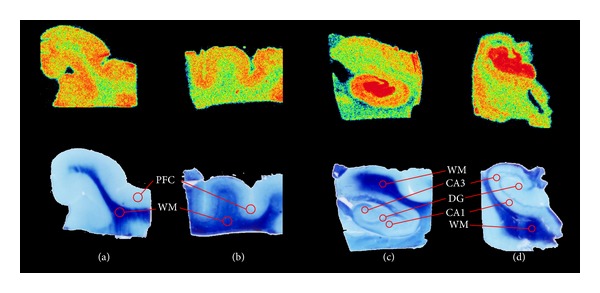
Example of tissues from the prefrontal cortex ((a), (b)) and hippocampus ((c), (d)) from control ((a), (c)), and AD ((b), (d)) tissues showing autoradiography binding (above) and luxol fast blue & cresyl violet staining (below). Studied regions of interest are the cortex, CA1, CA3, and Dentate Gyrus (DG). White matter (WM) was used as baseline.

The optimal conditions for the *in vitro* autoradiography binding techniques such as incubation time, washing procedure, and time of exposition to the phosphor imaging plate were obtained after preliminary experiments (data not shown). On the day of synthesis, sections were warmed to room temperature, air-dried, then preincubated in a 0.1 M phosphate-buffered saline solution (pH = 7.6) for 20 minutes in order to remove endogenous ligands. Tissue was then air-dried once more and incubated with [^18^F]FEOBV at 6.97 MBq/L in the same buffer solution at room temperature for 20 minutes. After incubation, slides were dipped three times in distilled water (4°C) and dried under a stream of cool air. The sections were then exposed on a radioluminographic imaging plate (Fujifilm BAS-MS 2025) for 20 minutes (see Figure 1 for examples). Autoradiography calibration was performed using 9 concentrations of [^18^F]- ranging from 0.03 to 7.72 MBq/L in the same imaging plate. The concentration used correlated linearly with the measured photostimulated luminescence unit per mm^2^ (*R* ^2^ = 0.9991, *P* < 0.0001). Corrected for time at the end of exposure, 1.32 MBq of [^18^F]FEOBV was used (specific activity of 22.93 TBq/mmol) in a solution of 3.77 MBq/L, for an effective concentration of 164.41 pM. 

 For histological staining of contiguous slices, a combination of luxol fast blue and cresyl violet staining was used [19]. Stained sections were digitized, and a total of five ROIs were drawn manually by an experimenter blind to group distribution: CA1, CA3, dentate gyrus (DG), PFC, and white matter as a baseline level. The drawn ROIs were then transposed to the corresponding autoradiography images. Activity in photostimulated luminescence unit per mm^2^ was calculated for each ROI using ImageGauge 4.0 (Fujifilm). To correct for background noise and nonspecific activity, results were normalized by the adjacent white matter values (e.g., [20]), since immunochemistry studies do not report significant levels of VAChT in the white matter [21, 22]. Group differences were then investigated using a *t*-test with Welch correction for heteroscedasticity between control and AD tissues. 

## 3. Results

There were no significant differences between control and AD groups in terms of sex distribution (*χ* ^2^ = 0.001, *P* = 0.971), age (*t*(31) = 0.302, *P* = 0.765), and postmortem delay (*t*(31) = 0.203, *P* = 0.840). On average, total brain mass was 12.4% lower in the AD group (*t*(30) = 2.84, *P* = 0.008) (see Table 1). For [^18^F]FEOBV binding parameters, all investigated grey matter ROIs showed higher binding than in the white matter baseline. In controls, the ROI-to-baseline binding ratios reached 1.82 ± 0.3 in the cortex, 1.85 ± 0.25 in CA1, 2.36 ± 0.64 in CA3, and was highest in the DG at 3.47 ± 0.55.

**Figure 2:**
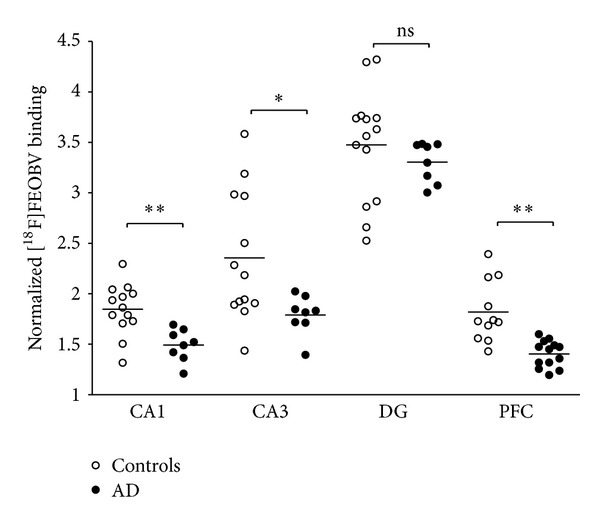
Ratio of [^18^F]FEOBV binding to adjacent matter for four ROIs. Difference between AD patients and controls subjects are present in CA1, CA3, and PFC. *P = 0.005, **P = 0.0005, ns: no significant difference.

In AD tissues, a 33% loss was observed in the PFC (*t*(13) = 4.2, *P* = 0.0005). Significantly lower binding was also observed in CA1 and CA3, with respective losses of 20% (*t*(18) = 3.93, *P* = 0.0005) and 25% (*t*(15) = 2.97, *P* = 0.005). AD tissues did not differ from controls in the DG (Figure 2). Neither age nor brain mass significantly predicted [^18^F]FEOBV binding in any region for either group. 

## 4. Discussion

 In summary, this study reports for the first time reduced VAChT availability in both the Nucleus Basalis of Meynert and medial septum projection areas in AD brain tissues. Reduced [^18^F]FEOBV binding observed in the AD neocortex as well as most of the hippocampus suggests a comparable degree of denervation between cortical and allocortical brain regions. Indeed, these finding mostly reflects the degeneration of these cholinergic pathways in AD [2, 23]. 

 A property of the radioligand saturation curve is that very low (tracer) concentrations follow a linear relationship with the receptor occupancy percentage at equilibrium. Here, 164.41 pM of [^18^F]FEOBV was used, which is less than 5% of the *K*_*d*_ of 4.1 nM for vesamicol [24] or 4.6 nM for another benzovesamicol analog [25]. Consequently, the magnitude of normalized binding differences observed here can be considered to be a direct representation of VAChT receptor density alterations in AD.

The magnitude of VAChT observed here in AD decreases (33% in PFC, 20% in CA1, and 25% in CA3) and is modest compared to previous cholinergic quantifications conducted with antibody-based techniques, such as immunohistochemistry or immunoblotting. When measured with Western blotting, VAChT levels in the PFC of AD patients have been reported to be 54% lower than those of controls [26]. Similarly, ChAT activity decreases in AD samples were described to range between 49% and 61% in various cortical and hippocampal regions [27]. Differences between autoradiography and immunohistochemistry have been frequently described and might reflect multiple factors including changes of radioligand affinity in the clinical population (e.g., [28]) or low radioligand specificity. 

In contrast to CA1 and CA3, the DG of the hippocampus appeared spared by cholinergic loss. This preservation of cholinergic terminals may be to do with the observation that the DG seems resistant to the glutamatergic neurodegeneration affecting both CA1 and CA3 in preclinical AD [29]. Invariant VAChT availability in the DG could thus reflect either sprouting of presynaptic axons or VAChT upregulation (e.g., [30, 31]) following initial losses. A unique resistance to such losses could also be specific to this region.

In terms of ROI distribution, the relative concentrations of [^18^F]FEOBV binding in the cortex and hippocampus match those found in postmortem studies using cholinergic markers both in humans [32, 33] and rodents [34]. Interestingly, the variance of [^18^F]FEOBV binding is much lower in the AD tissues than in controls both for the PFC and hippocampal ROIs. For instance, the CA3 coefficient of variance reached 27% in the control group, while it was only 11% in AD tissues. This represents a floor effect in the AD tissues, where most samples have minimal concentrations of VAChT terminals left from the affected afferents. Control samples, on the other hand, presented more variance, representing a wide spectrum of VAChT integrity normal in this age range.

In these same regions, 20 to 40% of control subjects have [^18^F]FEOBV binding values that overlap with those of AD patients. While this may be seen as a poor [^18^F]FEOBV sensitivity to differentiate AD from healthy controls, it remains consistent with the observation that AD pathological hallmarks (such as subclinical amyloidosis) are found in a substantial portion of aged asymptomatic individuals. Indeed, we have previously observed a significant reduction of hippocampal [^18^F]FEOBV binding in a rodent model of normal aging [14]. Here, the binding variance in control individuals does not appear to be explained by age, but this is likely due to the lack of younger control subjects in the sample.

 Cortical decreases in VAChT activity have been observed *in vivo* in AD, using Single Photon Emission Computed Tomography vesamicol analogs such as [^123^I]Iodobenzovesamicol [35]. Our results showed that PET radiomarker such as [^18^F]FEOBV might be useful also in quantifying such a VAChT reduction, while at the same time providing a better imaging resolution and faster kinetics. Additionally, while amyloid markers are currently considered the most sensitive for early detection [36], quantification of cholinergic markers tend to better describe a specific neurodegenerative event which closely correlate with clinical manifestations of AD [37, 38].

The present study must take into consideration methodological limitations. For instance, utilization of an autoradiography single-concentration protocol, as opposed to a quantitative receptor autoradiography approach, prevents the estimation of more detailed information regarding absolute kinetic properties of [^18^F]FEOBV. In addition, sample sizes were relatively small for drawing definitive conclusions, and the AD group was not controlled for neurofibrillary tangles accumulation (e.g., Braak stages). Other studies would be necessary to clarify the role of age in the [^18^F]FEOBV binding overlap between AD patients and control subject; younger control subjects would be useful in this respect. Moreover, tissues from individuals with Mild Cognitive Impairment would be most useful in providing the complete picture regarding VAChT availability in populations at risk. 

In conclusion, the present results support the notion that central cholinergic projections display discrete sites of specific vulnerability and resistance to AD. Furthermore, the present autoradiography study in human supports the use of [^18^F]FEOBV as a molecular probe to quantify selective deficits in cholinergic neurotransmission in AD. 

## Figures and Tables

**Table 1:** Subject data for AD patients and controls. There is no significant group difference between controls and AD patients for gender distribution, age, or postmortem delay.

Group	Sex	Age	PMD (h)	Brain wt (g)	ApoE status
AD	M	67	96	1410	3-3
AD	M	92	13.25	1200	3-3
AD	M	66	8.5	1075	4-3
AD	M	67	10.5	1235	3-3
AD	F	77	11.25	855	3-3
AD	M	76	24	1005	4-3
AD	F	74	18	1080	3-3
AD	M	73	26.25	1120	3-3
AD	M	63	21	1200	3-3
AD	F	88	24.5	955	3-3
AD	M	79	19.25	N/A	4-3
AD	M	79	12.75	1135	3-3
AD	M	79	24.75	1210	3-3
Control	M	59	29	1650	3-3
Control	M	60	7.25	1350	3-3
Control	M	65	28.5	1435	3-3
Control	F	74	11	1365	3-3
Control	M	71	76	1250	4-3
Control	M	67	24.75	1300	4-3
Control	M	79	22	1350	3-3
Control	M	70	32.75	1320	3-3
Control	F	90	25.5	1050	3-3
Control	M	91	6.75	1070	3-3
Control	M	68	18.5	1445	4-3
Control	M	80	13	1305	3-3
Control	F	91	26.5	1000	3-3
Control	F	79	17.75	1150	3-3
Control	F	95	23.75	1160	3-3
Control	M	85	26.75	1185	3-3
Control	M	88	8	1150	3-3
Control	F	51	26.25	1336.1	3-3
Control	M	61	8.75	1337.3	3-3
Control	M	59	17.67	1442.5	3-3
AVG (AD)	**77%** **Male**	**75.38**	**23.85**	**1123.33**	**23%** **“4-3”**
AVG (Controls)	**70%** **Male**	**74.15**	**22.52**	**1282.55**	**15%** **“4-3”**

## Abbreviations

AD: Alzheimer's disease
ChAT: Choline acetyltransferase
DG: Dentate gyrus
[^18^F]FEOBV: [^18^F]Fluoroethoxybenzovesamicol
PFC: Prefrontal cortex
ROI: Region of interest
VAChT: Vesicular acetylcholine transporter.
